# Morphological spectrum of lymphadenopathy in AIDS patients

**DOI:** 10.1186/1471-2334-14-S2-P56

**Published:** 2014-05-23

**Authors:** Kanthilatha Pai, Ranjini Kudva

**Affiliations:** 1 Kasturba Medical College, Manipal, India

## Introduction

Lymph node biopsy is essential in the evaluation of lymphadenopathy in patients infected with Human immunodeficiency virus to diagnose the many complications and institute proper treatment. Lymph node abnormalities in patients can be varied, showing spectrum of morphologic changes ranging from reactive lymphadenitis, opportunistic infections, Kaposi’s sarcoma, malignant lymphomas.

## Materials and methods

This is a retrospective study that analyses the morphological diagnosis of lymphadenopathy in patients with retroviral illness over a 2 year period (2012-2013). All lymph node biopsies performed in HIV positive patients for work-up of lymphadenopathy are included and the following clinical information is analyzed: Age, sex, clinical symptoms, duration of illness, retroviral treatment and CD4 counts. The morphological diagnosis is classified into reactive lymphadenitis which is further divided into Pattern A, B and C, opportunistic infections, lymphomas and others.CD4 levels and other parameters are analyzed in the study.

## Results

A total of 29 patients with retroviral illness in whom lymph node biopsies were done were included in the study, of which 17 were males and 12 were females. The most common age group ranged from 31-40 years in both males and females. Fever and generalized lymphadenopathy were the most common symptoms .Reactive lymphadenitis and opportunistic infections were diagnosed in 11 patients each (42.3%) showing, Pattern A in 7 patients and Pattern B in 4 patients. Tuberculosis was the most frequent opportunistic infection in 9 patients, followed by Crytococcal infection in 2 patients. 4 patients with Non-Hodgkins lymphoma and 1 patient with polymorphous Hodgkin like B cell lymphoproliferative disorder was diagnosed. 2 cases included Lympho-epithelial cyst and infarction of lymph node.CD 4 counts were available for 16 patients which was low in all patients and ranged from 21-181/cumm.

## Conclusion

It is imperative to perform lymph node excision in HIV patients as it can display wide variety of conditions ranging from reactive to opportunistic infections to neoplastic conditions, which require specific treatment. Lymph node biopsy in HIV patients will help to understand the epidemiology and geographical prevalence of lymph node abnormalities in HIV patients.

